# A Systematic Review of the Protective Actions of Cat’s Whiskers (Misai Kucing) on the Central Nervous System

**DOI:** 10.3389/fphar.2020.00692

**Published:** 2020-05-13

**Authors:** Yin-Sir Chung, Brandon Kar Meng Choo, Pervaiz Khalid Ahmed, Iekhsan Othman, Mohd. Farooq Shaikh

**Affiliations:** ^1^Neuropharmacology Research Strength, Jeffrey Cheah School of Medicine and Health Sciences, Monash University Malaysia, Bandar Sunway, Malaysia; ^2^Liquid Chromatography-Mass Spectrometry (LCMS) Platform, Jeffrey Cheah School of Medicine and Health Sciences, Monash University Malaysia, Bandar Sunway, Malaysia; ^3^School of Business, Monash University Malaysia, Bandar Sunway, Malaysia; ^4^Global Asia in the 21^st^ Century (GA21), Monash University Malaysia, Bandar Sunway, Malaysia

**Keywords:** *Orthosiphon stamineus*, central nervous system, neuroprotection, neurotoxicity, oxidative stress

## Abstract

*Orthosiphon stamineus* (OS) or *Orthosiphon aristatus* var. *aristatus* (OAA) is commonly known as cat’s whiskers or “misai kucing”. It is an herbaceous shrub that is popular in many different traditional and complementary medicinal systems. Its popularity has been justified by the plethora of studies that have shown that the secondary metabolites of the plant has effects that range from anti-inflammatory and gastroprotective to anorexic and antihypertensive. As such, OS could also be a potential treatment for Central Nervous System (CNS) disorders. However, a cohesive synthesis of the protective actions of OS was lacking. This systematic review was therefore commenced to elaborate on the various protective mechanisms of OS in the CNS. The PRISMA model was used and five databases (Google Scholar, SCOPUS, SpringerLink, ScienceDirect, and PubMed) were searched with relevant keywords to finally identify four articles that met the inclusion criteria. The articles described the protective effects of OS extracts on Alzheimer’s disease, epilepsy, learning and memory, oxidative stress, and neurotoxicity. All the articles found were experimental or preclinical studies on animal models or *in vitro* systems. The reported activities demonstrated that OS could be a potential neuroprotective agent and might improve CNS conditions like neurodegeneration, neuroinflammation, and oxidative stress.

## Introduction

Neuroprotection is a relatively new concept in neuroscience research, coined to incorporate a great variety of mechanisms that aim to prevent neuronal injury and loss of various brain functions with an ultimate goal to better preserve brain function (Schapira, 2010). Thus, neuroprotection is being explored as a possible treatment strategy for Central Nervous System (CNS) disorders such as neurodegeneration, stroke, or trauma that result in CNS injuries (Lalkovičová and Danielisová, 2016). These disorders may occur through a wide variety of mechanisms, although some common themes include abnormal protein behavior, oxidative stress, mitochondrial dysfunction, neuroinflammation, excitotoxicity, and others (Jellinger, 2010). These varied mechanisms result in an equally varied array of disorders such as epilepsy, motor neuron disease, Parkinson’s disease, multiple sclerosis, and Alzheimer’s disease (Vajda, 2004). Neurological disorders remain as one of the greatest threats to public health. There are several gaps in understanding the many issues related to neurological disorders, but we already know enough about their nature and treatment to be able to shape effective intervention responses to some of the most prevalent among them.

In the search for novel CNS treatments, medicinal plants are worthy of attention as they have been used as natural remedies since the dawn of civilization due to their substantial protective effects on human health. Thus, the different traditional medicinal systems practiced by the diverse communities worldwide may contain clues pointing towards natural remedies or an effective cure. Among these systems are the Sowa Rigba in Bhutan, Jamu in Indonesia, Ayuverda and Unani in India, Bangladesh, Nepal and Sri Lanka, homeopathy practices in European societies, Islamic Traditional Medicine among Muslims, Traditional Chinese Medicine among the Chinese, and Koryo Medicine among the Koreans (World Health Organization, 2013). Even today, medicinal plants still have significant roles to play in not only addressing various health issues, but also in supporting a healthy lifestyle. In modern medicine, extensive research evidence has shown that many plant-derived secondary metabolites have notable protective effects on the CNS (Edeoga et al., 2005; Rabiei, 2017; Manchishi, 2018). For instance, discovered in the 1940s, reserpine which is an indole alkaloid (Gurib-Fakim, 2006; Abdelfatah and Efferth, 2015) isolated from the roots of *Rauwolfia serpentina* (Indian snakeroot) was an antipsychotic used to treat schizophrenia in the past (Nur and Adams, 2016) and has heralded the beginning of a new era of drug treatment for mental disorders. Cannabis-based products such as cannabidiol (CBD) is another candidate in the spotlight for its efficacy and safety for treating different forms of epilepsy (See completed cannabidiol clinical trials in epilepsy https://clinicaltrials.gov/) (Perucca, 2017; Silvestro et al., 2019).

*Orthosiphon stamineus* (OS) or *Orthosiphon aristatus* var. *aristatus* (OAA), is commonly known as cat’s whiskers or “misai kucing”. Belonging to the *Lamiaceae* family, it is a perennial, herbaceous medicinal shrub that stands 30 to 150 cm tall and is ubiquitous in the temperate and tropical areas of Asia, Australia, and the Pacific. The stamens and pistil can grow as far as 2 cm beyond the flower clusters during full bloom to form a shape that is reminiscent of cat whiskers (Sawaya and Poupelloz, 2011; Ameer et al., 2012; Adnyana et al., 2013). OS is believed to originate in South East Asia and is unsurprisingly known by a myriad of local names such as java tea (English common name), “neko no hige” (Japan), “mao xu cao” (China), “se-cho” or “myit-shwe” (Myanmar); “rau-meo” (Vietnam) and “yaa-nuad-maew” or “pa-yab-mek” (Thailand), “misai kucing”, “ruku hutan”, or cat’s whisker (Malaysia), “kumis kucing”, “kutum”, “mamam”, “bunga laba-laba”, “remuk jung/remujung”, “songot koceng” and “sesalaseyan” (Indonesia); “kabling gubat/kabling parang” (Philippines). It is a popular medicinal plant due to its widespread and prolonged used in many traditional and complementary medicinal systems across many South East Asian and European countries for the prevention and treatment of disorders such as rheumatism, diabetes, hypertension, and epilepsy among many others (Sawaya and Poupelloz, 2011; Adnyana et al., 2013). OS has been reported to be anorexic (Son et al., 2011), diuretic, hypouriceamic and antiurolithic; anti-inflammatory, analgesic and antipyretic; antioxidative, hepatoprotective, nephroprotective, gastroprotective, cardiovascular-protective, hypolipidaemic, antihypertensive and anti-obesity; hypoglycaemic, antiproliferative, cytotoxic and anti-angiogenic; antimicrobial and also has anti-sebum activity (Ameer et al., 2012; Adnyana et al., 2013). Given its traditional use as a treatment for epilepsy (which is a CNS disorder) and that the ability of OS to counteract oxidative stress and inflammation [which are both implicated in CNS disorders (Kamat et al., 2008; Amor et al., 2010)], OS could have the potential to be neuroprotective. However, while there are extensive reviews of OS, both narrative and systematic, reviews on neuroprotective potential of OS was however found to be lacking. Hence, a comprehensive systematic literature review was commenced to address this shortcoming and to elaborate on the protective actions of OS on the CNS.

## Materials and Methods

### Search Method

Five databases (Google Scholar, SCOPUS, SpringerLink, ScienceDirect, and PubMed) were searched to identify relevant articles using the keywords “*Orthosiphon stamineus* AND Brain Protection” and “*Orthosiphon stamineus* AND CNS”. The common term *Orthosiphon stamineus* was used as the alternative name for *Orthosiphon aristatus* as this term typically produces more search results. The results were then filtered to include only studies between January 2009 and December 2019 to maximise the inclusion of more recent publications in this review, while minimizing the possibility of inadvertently excluding older studies. SCOPUS and ScienceDirect results were exported as RIS files, Google Scholar results were exported using Harzing’s Publish or Perish 7 into RIS files, SpringerLink results were individually exported as RIS files and PubMed results were exported as nbib files. All the exported files were then imported into EndNote X9.2 to generate a library, which was then exported as a text file in the EndNote Export style. The text file was then imported into Rayyan (Ouzzani et al., 2016) and the generated list of unique entries (software identified duplicates were automatically excluded) were screened for their relevance based on their title and abstract.

### Study Selection and Inclusion Criteria

Only original research articles were considered for their content for this systematic review as other publication types would not have provided sufficient information for evaluation and comparison. Any duplicated results that were missed by Rayyan were also removed, as well as those that have no relevance to OS or the field of neuroscience. The result selection process was conducted as per the PRISMA guidelines (Moher et al., 2015).

## Results

**Figure 1:**
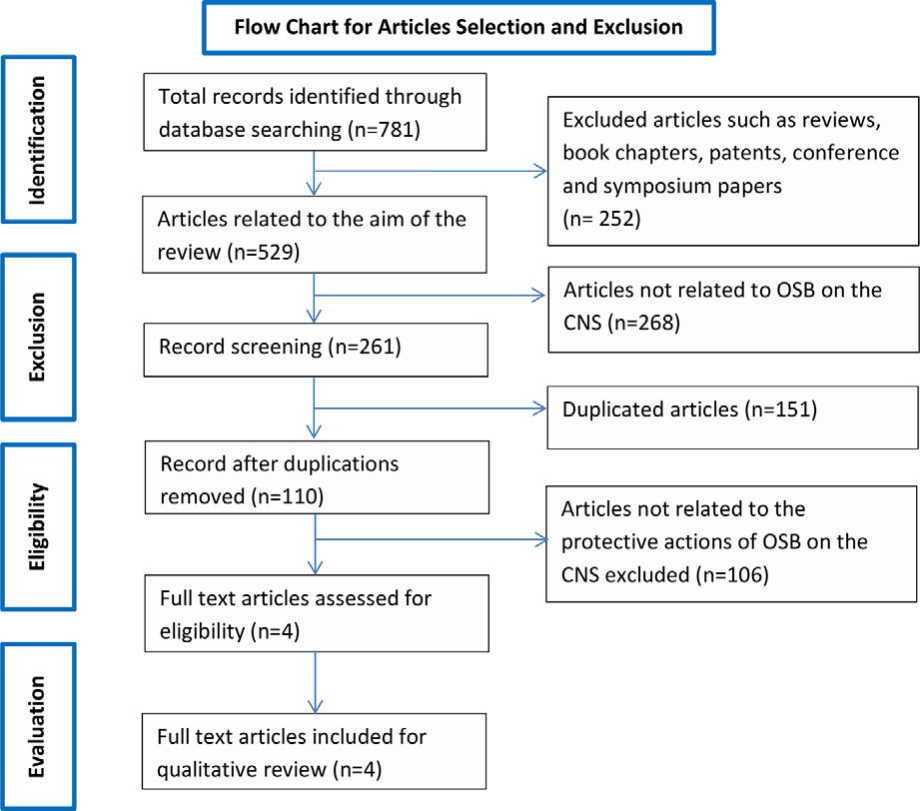
Flow chart of the study selection criteria based on the Preferred Reporting Items for Systematic Reviews and Meta-Analyses (PRISMA) guidelines.

Searching the aforementioned databases using the chosen keywords resulted in a total of 781 records. Of the 781 records, 737 records were from Google Scholar, 0 from SCOPUS, 20 from SpringerLink and 24 Science Direct and 0 from PubMed. After applying exclusion criteria, 777 records were removed, which includes 101 duplicates and 676 articles not related to the scope of the review (**Figure 1**). The remaining four records underwent full text evaluation and no further records were removed as all four records were found to be relevant. The four articles are summarized in **Table 1** and discussed in the present systematic review.

**Table 1:** Summary of studies reported for neuroprotective effects of *Orthosiphon stamineus* Benth.

Study details	Study design and model	Testing dose	Active constituents	Administration route	Pharmacological actions	Summary of findings
Retinasamy et al. (2019)	*in vivo* (adult Sprague Dawley rats)	50, 100 and 200 mg/kg	Not mentioned	Oral and intraperitoneal (i.p., at a volume corresponding to 0.1 mL/100 g of b.w.)	Anti-AD *via* the BDNF-TrKB and CREB-BDNF pathways and can promote hippocampal neurogenesis	Ethanolic extract of OS might be a promising candidate as a memory enhancer or as a therapeutic treatment for neurodegenerative diseases like AD
Choo et al. (2018)	*in vivo* (adult *Danio rerio*)	50, 100 and 200 mg/L	Not mentioned	Oral	Anti-convulsive and anti-inflammatory *via* TNFα pathway	Ethanolic leaf extract of OS has the potential to be a novel symptomatic treatment for epileptic seizures as it is pharmacologically active against seizures in the zebrafish
George et al. (2015)	For *in vitro* adenosine receptors A2_A_ and A_1_ assays: human recombinant HEK-293 cells and Wistar rat vas deferens For *in vivo* work: adult male Sprague Dawley (3-mo, 200 – 250 g) and juvenile male SD rats (35–40-do, 75 –100 g)	A2_A_ binding assay: 15 and 150 µg/mL; A2_A_ and A_1_ functional assay antagonist and agonist: 3, 30 and 300 µg/mL 200, 300 and 600 mg/kg (p.o.) 60 and 120 mg/kg (i.p.)	ombuin (3,3′,5-trihydroxy-4′,7-dimethoxyflavone) (0.14%),3′-hydroxy-4′,5,6,7-tetramethoxyflavone (0.10%), sinensetin (0.07%), orthosiphol B (0.26%), orthosiphol A (0.67%), staminol A (0.45%), orthosiphonone A (0.12%)	Oral and intraperitoneal	Enhanced learning and memory *via* blockade of receptors A_1_ and A_2A_	Standardised ethanolic extract of OS may reverse age-related deficits in short-term social memory and can be considered to prevent or decrease the rate of neurodegeneration
Sree et al. (2015)	*in vitro* (human neuroblastoma cell line, SH-SY5Y)	0.01 to 1 mg/mL	Not mentioned	N/A	Antioxidative, antiapoptotic and neuroprotective to alleviate ROS-mediated dysfunction of dopamenergic neurons and neuronal cell death	OS methanol bioactive guided fraction (OMF) can attenuate the H_2_O_2_ induced oxidative stress by improving the antioxidant status, cell viability, ROS formation, mitochondrial membrane integrity and regulation of gene expression in the neuronal cells. OMF can be considered as an alternative to some of the toxic synthetic antioxidants which are used in food, cosmetics and pharmaceutical applications

AD, Alzheimer’s Disease; BDNF, brain-derived neurotrophic factor; b.w., body weight; CREB, cAMP response element binding protein; do, day-old; i.p., intraperitoneal; mo, month-old; OS, Orthosiphon stamineus; p.o., per oral; SD, Sprague Dawley; TrKB, Tropomyosin receptor kinase B.

**Figure 2:**
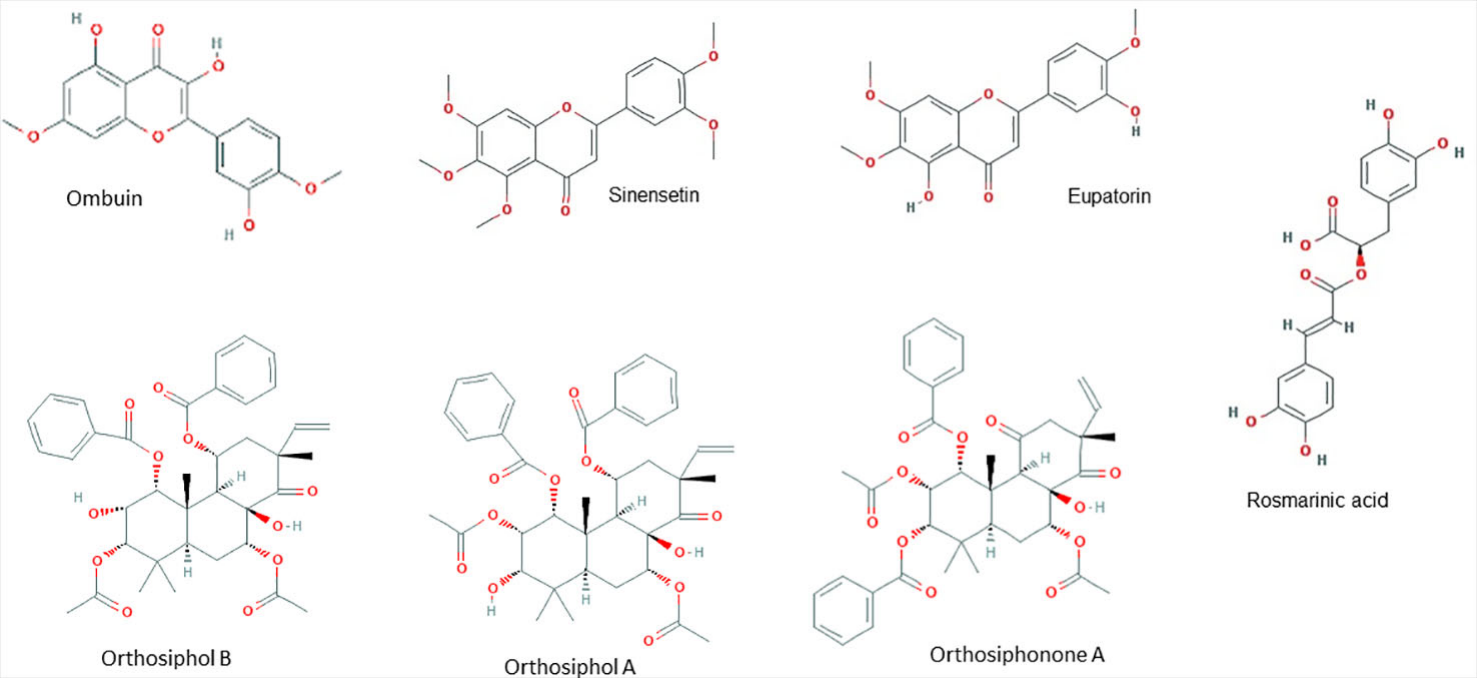
Principal chemical constituents of *Orthosiphon stamineus*.

HPLC analysis of an ethanolic OS extract for seven reference compounds by George et al. (2015) showed that their standardised extract contained ombuin (3,3′,5-trihydroxy-4′,7-dimethoxyflavone) (0.14%), 3′-hydroxy-4′,5,6,7-tetramethoxyflavone (0.10%), sinensetin (0.07%), orthosiphol B (0.26%), orthosiphol A (0.67%), staminol A (0.45%), and orthosiphonone A (0.12%) as shown in **Figure 2**. A summary of the preparations used in each of the selected articles is given in **Table 2**.

**Table 2:** Summary table of the preparations used by the studies in the present systematic literature review.

Study	Formulation	Source	Species, concentration	Quality control reported? (Y/N)	Chemical analysis reported? (Y/N)
Retinasamy et al. (2019)	50% ethanolic extract	NatureCeuticals Sdn Bhd, Kedah DA, Malaysia	*Orthosiphon stamineus*, Unknown concentration	Y- Prepared in a Good Manufacturing Practice (GMP) based environment	N
Choo et al. (2018)	50% ethanolic extract	NatureCeuticals Sdn Bhd, Kedah DA, Malaysia	*Orthosiphon stamineus* Unknown concentration	N	N
George et al. (2015)	70% ethanolic extract	Prepared by George et al. (2015)	*Orthosiphon stamineus* [Biotropics Malaysia Berhad, Malaysia], 1000g	Y - Plant material identified by a taxonomist on the basis of exomorphic characters and literature review	Y - HPLC
Sree et al. (2015)	Methanolic fraction	Prepared by Sree et al. (2015)	*Orthosiphon stamineus* [Western Ghats, India], 3 mg/m	Y - Plant material authenticated at Acharya Nagarjuna University, Guntur, India	Y - HPLC

### OS and Alzheimer’s Disease

Alzheimer’s disease (AD) is an age-related neurodegenerative disorder that leads to cognitive, functional and behavioral alterations (Cummings et al., 2018). The cause of Alzheimer’s disease is currently unknown, but is believed to result from a variety of factors such as an abnormal accumulation of beta-amyloid, death of cholinergic neurons, aggregation of microtubule tau proteins, metal dyshomeostasis, and metal-induced oxidative stress (Simunkova et al., 2019). As compared to rats given only 1 mg/kg of scopolamine, an ethanolic extract of OS has been shown by Retinasamy et al. (2019) to enhance memory when given to rats in a scopolamine-induced amnesia model which mimics the memory impairment in human Alzheimer’s patients. They believed that the effects of the extract could be due to its positive modulation of the *CREB1* (cAMP response element-binding protein), *BDNF* (Brain-derived neurotrophic factor), and *TrKB* (Tropomyosin receptor kinase B) genes which are involved in memory, as BDNF binds to the TrKB receptor with a high affinity.

### Epilepsy

Epilepsy is a CNS disorder that afflicts approximately one in every hundred people, with about half of those cases being idiopathic (Holland, 2014). Epileptic seizures are a short-term appearance of various signs and/or symptoms due to unusually excessive or concurrent brain activity (Fisher et al., 2014). Seizures can affect memory, cognition, behavior, or emotional state among others, though not always in tandem (Fisher et al., 2005). As compared to zebrafish given 170 mg/kg of the pro-convulsant pentylenetetrazol only, OS extract has been shown by Choo et al. (2018) to have anti-seizure properties in an adult zebrafish model of pentylenetetrazol induced acute seizures. Choo et al. (2018) remarked that OS extract appears to prevent the upregulation of NF-*kB* (Nuclear Factor kappa-light-chain-enhancer of activated B cells) and *NPY* (Neuropeptide Y), which was puzzling as the upregulation of both genes is associated with a decrease in seizure threshold (Lubin et al., 2007; Wickham et al., 2019). Choo et al. (2018) postulated that these two pathways were not activated in the presence of OS as it exerted its anti-seizure activity *via* one or more other mechanisms. They suggested that one of these mechanisms could be at least partially due to the downregulation of *TNFα* (Tumor Necrosis Factor alpha).

### Learning and Memory

In the CNS, endogenous adenosine receptors have been associated with an array of functions including sleep and arousal, cognition, learning and memory, protection from neuronal damage and degeneration as well as influencing neuronal maturation (Chen et al., 2007). Adenosine receptors (AR) belong to the G-protein-coupled receptor family (Fredholm et al., 1999) and their antagonism can produce CNS-enhancing effects. Adenosine is known to modulate cognitive functions through the adenosine receptors A_1_ (A1R) and adenosine A_2A_ (A2AR). The selective blockade of A1R and A2AR can facilitate learning and memory in *in vivo* models (Pereira et al., 2002; Takahashi et al., 2008). A proprietary standardized ethanolic OS leaf extract has been shown to have memory-enhancing properties in Sprague Dawley rats possibly by reversing age-related deficits in short-term social memory and due to involvement of adenosine A_1_ and adenosine A_2A_ as a target bioactivity site in the restoration of memory (George et al., 2015). They determined this *via in vitro* binding assays (A2A binding assay, A1 and A2A functional agonist and antagonist activity assays) which showed that an OS concentration of 150 µg/ml was sufficient to produce 74% inhibition of the A2AR and 300 *µ*g/ml essentially produced complete inhibition of the A1R and A2AR. They measured social memory *via* the social recognition tests which compares the time spent by an adult rat in investigating a juvenile rat, with the assumption that a second successive encounter should produce a shorter investigation time if the juvenile rat is recognised by the adult. By comparing the successive investigation times before and after treatment with OS, George et al. (2015) concluded that the standardized ethanolic extract of OS can be considered to prevent or to decrease the rate of neurodegeneration as compared to a rats given the vehicle control of distilled water.

### Oxidative Stress-Induced Neurotoxicity

Oxidative stress has been shown to play a key role in regulating redox reactions in the CNS. Elevated levels of oxidative stress as a result of increased generation of free radicals such as reactive oxygen species (ROS), have been linked to apoptosis in neuronal cells which in turn leads to various neurological disorders (Hensley et al., 2000). As compared to an unspecified vehicle control, a methanolic fraction extracted from OS leaves (OMF) has been shown by Sree et al. (2015) to have neuroprotective and cytoprotective effects in the human SH-SY5Y cell model. When 1,000 µg of OMF was given to SH-SY5Y cells, H_2_O_2_-induced oxidative stress (increased cell viability, decreased ROS formation, and lipid peroxidation) and the loss of mitochondrial membrane integrity (decreased amounts of lactate dehydrogenase leakage and increased mitochondrial membrane potential) was prevented. Lactate dehydrogenase leakage can also be indirectly used as a measure for cell death (Smith et al., 2011) and the decreased level found by Sree et al. (2015) also suggests that OMF is not cytotoxic to SH-SY5Y cells. SH-SY5Y cells given 1,000 µg of OMF also had improved antioxidant status (increased SOD, CAT and GPx expressions). Superoxide dismutase (SOD), catalase (CAT), and glutathione peroxidase (GPx) are active scavengers of superoxide (O_2_·^−^) and hydrogen peroxide (H_2_O_2_). Additionally, OMF enhanced the expression of neuronal biomarker genes (increased levels of *BDNF*, *TH*, and *AADC*) in SH-SY5Y cells. *BDNF*, *TH* (Tyrosine hydroxylase), and *AADC* (Amino acid decarboxylase) are genes that play a pivotal role in the survival and differentiation of dopaminergic neurons. This increase occurred both in the presence or absence of H_2_O_2_ oxidative stress, though to a greater degree when H_2_O_2_ was present, even though H_2_O_2_ alone downregulates these genes. Based on their findings, Sree et al. (2015) proposed that the neuroprotective potential of OMF could possibly be *via* antioxidative mechanisms.

## Discussion

**Figure 3:**
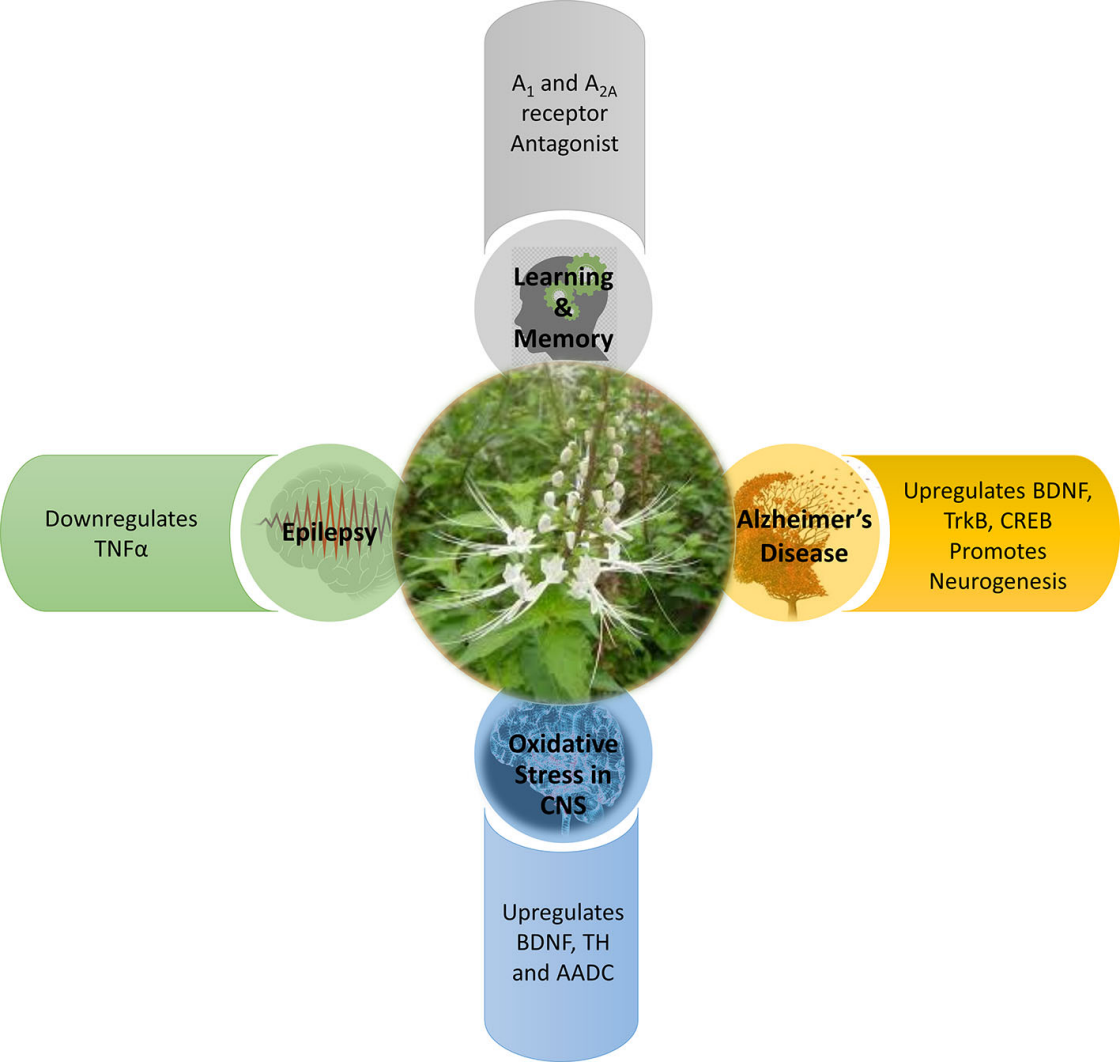
Mechanism of action of *Orthosiphon stamineus* in CNS disorders.

Various forms of CNS disorders may not be caused by only a single factor, but may also be a combination of multiple factors. For instance, the synaptic dysfunction and memory impairment detected in AD (McGeer and McGeer, 2010; Gomes et al., 2011) are also components commonly found in epilepsy (Wen et al., 2010; Holmes, 2015) and oxidative stress-induced neurological conditions (Aguiar et al., 2012; Ho et al., 2015; Chitnis and Weiner, 2017; Walker, 2018). There exists an interplay between oxidative stress, neurodegeneration, and neuroinflammation. The studies elaborated in this systematic review have collectively proposed that OS has a protective role to play in the CNS. OS has demonstrated its ability to intercept the cross-talk between neurodegeneration, neuroinflammation, and oxidative stress, and hence contributes to neuroprotection *via* four mechanisms: enhancing memory, anti-inflammation, anti-seizure, and antioxidative as depicted in **Figure 3**.

### Modulation of BDNF Signalling and Neurogenesis

All neurodegenerative processes are evolving conditions (Gomes et al., 2011). Based on the studies in this systematic review, OS contributes to the prevention of evolving memory impairment by modulating the BDNF-TrkB and CREB-BDNF signalling pathways as well as promoting neurogenesis. These three pathways serve to counteract the progressive impairment of memory as a result of neurodegeneration (Toledo and Inestrosa, 2010); with BDNF together with its major receptor TrkB being involved in synaptic plasticity in the form of long term potentiation and also long term memory formation and consolidation (Cunha et al., 2010). Modulation of these signalling pathways results in the initiation of three major signalling pathway cascades, namely phospholipase Cγ (PLCγ), phosphatidylinositol 3-kinase (PI3K), and extracellular signal-regulated kinases (ERK) (Cunha et al., 2010). All three of these pathways and the transcription factor CREB1 have been associated with learning and memory, though the exact role of BDNF and any interactions between these pathways have yet to be fully uncovered (Barco et al., 2003; Thomas and Huganir, 2004; Liu et al., 2006; Gruart et al., 2007). The upregulation of CREB also has positive effects on memory consolidation and performance as CREB modulates BDNF expression and thus upregulates it in turn (Suzuki et al., 2011). In addition, the transcription factor CREB, is also capable of promoting anti-inflammatory responses. These anti-inflammatory responses could be neuroprotective by inhibiting unwanted inflammation, tissue damage, and autoimmune response (Wen et al., 2010) in the CNS. Owing to the anti-inflammatory potential of OS, this may also enhance its capability to prevent neurodegeneration due to a decrease in CREB. OS extract could also reverse the scopolamine induced suppression of neurogenesis in the dentate gyrus of the hippocampus, which again plays a role in spatial memory. The role of the dentate gyrus in spatial memory is postulated to be in the alignment of an internally memorized spatial map with external landmarks as determined by sensory information and thus an impaired dentate gyrus would also impair spatial memory if the external landmarks change (Won Lee et al., 2009).

### Antagonism of A1R and A2AR

OS contributes to enhancing learning and memory by modulating the adenosine receptors, A1R and A2AR in the CNS, as discovered by George et al. (2015). In a human study using positron emission tomography (PET) and 8-dicyclopropylmethyl-1-[11C] methyl-3-propylxanthine, Fukumitsu et al. (2008) showed a significant reduction in A1R binding potential in the temporal cortex and thalamus of AD patients as compared with elderly normal subjects. Cunha (2005) also reported a decrease of A1R density and efficiency in neurodegenerative disorders. Canas et al. (2009) and Dall’lgna et al. (2003) have showed that A2AR antagonism prevents synaptic loss as well as neuronal death triggered by Aβ synthetic peptides and thus suggests that modulation of A2AR antagonism could have neuroprotective effects in AD. One interesting finding by Prediger and Takahashi (2005) is that antagonising either adenosine receptor, A1R or A2AR, can still result in an enhancement of social memory. An experiment by Kaster et al. (2015) proposed that caffeine acts through neuronal adenosine A2A receptors to prevent mood and memory dysfunction triggered by chronic stress. This finding may suggest that OS could be working in a similar manner. By acting on these adenosine receptors, synaptic degeneration and subsequent neuronal death can be improved. This enhances learning and memory to help in arresting neurodegeneration at the early stages (Gomes et al., 2011).

### Modulation of Dopaminergic Neurotransmission

OS contributes to the prevention of neurodegeneration by modulating dopaminergic neurotransmission (Sree et al., 2015). This suggests that OS could be preventing dopamine neuron dysfunction or loss as a consequence of neurodegeneration, oxidative stress, neuroinflammation, or a combination of the aforementioned. There is a large body of evidence that associates BDNF, TH, and AADC with the survival, differentiation, and regulation of dopaminergic neurons (Baquet et al., 2005; Bathina and Das, 2015; Lima Giacobbo et al., 2019). Additionally, OS may also be capable of enhancing memory, besides preventing memory loss. Dopamine together with noradrenaline, are two key enzymes in catecholamine neurotransmitter synthesis. Catecholamine neurotransmission is important as it is implicated in working memory performance (Cools and D’Esposito, 2011).

### TNFα Signalling

OS contributes to the prevention of seizures and epilepsy by downregulating *TNFα* (Choo et al. (2018). *TNFα* has been shown to play a part in not only systemic inflammation, but also in epilepsy (Rana and Musto, 2018) and oxidative stress (Aguiar et al., 2012). Sinha et al. (2008) have found a decrease in cytokines, including *TNFα*, in patients with epilepsy. An experiment by Ho et al. (2015) has also reported an increased seizure susceptibility due to the induction of neuroinflammation and oxidative stress in the hippocampus. Perhaps the anti-convulsive potential of OS could be acting against neuroinflammation owing to its anti-inflammatory properties (Yam et al., 2008; Yam et al., 2010). The downregulation of *TNFα* also reduces AMPA (α-amino-3-hydroxy-5-methyl-4-isoxazolepropionic acid) glutamatergic receptor trafficking in turn and hence decreases excitatory synaptic transmission (Patel et al., 2017). In addition, the downregulation of TNFα also decrease glutamate release through the downregulation of glutaminase and microglia gap junctions (Takeuchi et al., 2006) and also reduces the endocytosis of inhibitory γ-aminobutyric acid (GABA) receptors (Stellwagen et al., 2005).

### Redox Signalling

Oxidative stress, alone or in combination, plays a pivotal role in various CNS disorders and can also damage essential macromolecules (Čolak, 2008; Pizzino et al., 2017). For instance, post-mortem samples from patients with AD revealed that there were elevated markers of oxidative stress including protein carbonylation and lipid peroxidation within the tissue (Butterfield and Lauderback, 2002; Sultana et al., 2006). Based on the studies in this systematic review, OS has demonstrated its capability in modulating the redox signalling cascade by regulating SOD, CAT, and GPx, to scavenge ROS. These enzymatic antioxidants collectively known as the first line defense antioxidants (Ighodaro and Akinloye, 2018). They act very fast to suppress or prevent the formation of free radicals or reactive species in cells by neutralizing any molecule with the potential of developing into a free radical or any free radical with the ability to induce the production of other radicals. An experiment by Akowuah et al. (2005) has reported that an OS leaf extract containing major components including sinensetin, eupatorine, 3′-hydroxy-5,6,7,4′-tetramethoxyflavone, rosmarinic acid, and quercetin, shows significant free radical scavenging and antioxidant abilities. In another experiment by Yam et al. (2007), a standardized OS extract has also demonstrated anti-oxidant and free-radical scavenging abilities. With these science-backed evidence, OS has been suggested to play a fundamental role in the redox signalling cascade—by activating the first line enzymatic antioxidants defense, it is able to scavenge and prevent the accumulation of free radicals (pro-oxidants such as O_2_·^−^, H_2_O_2_ and hydroxyl, ·OH) and thus, reducing the deleterious effects of oxidative stress. OS can thereby contribute to the delay of the onset and progression of neurodegeneration such as neuronal death, AD, and dementia induced by oxidative stress (Uberti et al., 2002; Zhang et al., 2010; Barsukova et al., 2011).

In the face of increased oxidative stress, the inability of antioxidant defense systems to counter the proinflammatory response is key to the onset and progression of neurodegeneration and neuroinflammation (Bakunina et al., 2015). There exists large amount of evidence suggesting that several basic mechanisms which drive neurodegeneration may be triggered by inflammatory cells and their mediators at various stages of the neurodegenerative cascade (Wyss-Coray and Mucke, 2002; Chitnis and Weiner, 2017; Kinney et al., 2018; Walker, 2018). In AD, for instance, the two most common mechanisms are mitochondrial dysfunction and inflammation mediated by cytokines and activated immune cells. de la Monte and Wands (2006) found that the neurons of AD patients have a high percentage of damaged mitochondria, which may be due to the increased presence of mutations in the mitochondrial DNA. Oxidative damage of mitochondrial proteins and DNA is found even in early stages of AD, suggesting a role of oxidative stress in disease progression (Nunomura et al., 2001; de la Monte and Wands, 2006). In both astrocytes and microglia, toll-like receptor (TLR)-mediated activation can release cytokines and chemokines (i.e. TNFα, IL-1, -3, -6, CCL2/MCP-1) as well as ROS, which can either promote neuronal survival or, in case of massive damage as in AD (Lee et al., 2018), ischemia or spinal cord injury, may promote inflammation and aggravate neuronal damage (van Noort and Bsibsi, 2009; Hinojosa et al., 2011). Activated immune cells, particularly macrophages, can produce ROS which also contributes to mitochondrial dysfunction and ultimately neuronal apoptosis (Chitnis and Weiner, 2017). In two separate experiments by Yam and colleagues (Yam et al., 2008; Yam et al., 2010), the components of OS leaves which are responsible for its anti-inflammatory effect were found to be the polymethoxylated flavones sinensetin, eupatorine and 30-hydroxy-5,6,7,40-tetramethoxyflavone.

### Safety and Toxicity of OS

According to the Assessment Report on *Orthosiphon stamineus* Benth. (EMA/HMPC/135701/2009), clinical safety data is limited. The report concludes that no safety problems concerning the traditional use of OS or its preparations overall. OS preparations are considered not harmful when used in the recommended dosages for specified preparations. Its use however, can only be limited to the adults and elderly as no data on the use in children and adolescents is available. As there is no information on reproductive and developmental toxicity, its use during pregnancy and lactation cannot be recommended.

### Pharmacokinetics of OS

Recently, selected secondary metabolites of OS have been investigated for their pharmacokinetics. Guo et al. (2019) studied the plasma pharmacokinetics of an OS extract with selected nine analytes (protocatechuic acid, PCA; danshensu, DSS; caffeic acid, CAA; rosmarinic acid, RA; sinensetin, SIN; eupatorine, EUP; cichoric acid, CA; salvianolic acid A, Sal A and salvianolic acid B, Sal B) in rats after oral administration at 10 g/kg. The maximum plasma time (T_max_, h) ranged between 0.36–2.79 and the maximum concentration (C_max_, ng/ml) was between 2.05–1008.02. The half-life (t_1/2z_, h) ranged from 0.59-13.50, area under the plasma concentration versus time curve from zero to last sampling time (AUC_0–t_, h) was 1.66–9421.62 and mean residence time (MRT_0–t_, h) was 0.79–9.02. In another study by Shafaei et al. (2017), the plasma pharmacokinetics of an ethanolic OS extract with four marker compounds (RA, SIN, EUP and 3′-hydroxy-5,6,7,4′-tetramethoxyflavone, TMF) in rats after oral administration at 1,000 mg/kg and intravenous administration at 250 mg/kg *via* the tail vein was elucidated. After oral administration, T_max_ was ranged between 2.83–3.17 h and C_max_ was between 0.77–1.89 µg/ml, AUC (0–24, µg h/ml) ranged between 0.91–4.37 and bioavailability was between 15.28–26.45%. After IV administration, the estimated volume of distribution (V_d_, l/kg h) ranged between 0.09–1.06, mean clearance value (CL, l/kg h) was 0.12–0.66, elimination rate constant (K_e_, h-1) was 0.62–1.44, biological half-life (t_1/2_, h) 0.49–1.13 and AUC (24-∞, µg h/ml) ranged between 1.73–4.13. These studies suggested that different OS extracts display a varying pharmacokinetic profiles dependent upon the route of administration.

### Future Directions

All the four articles discussed in this review share one major limitation, that is all the studies were in the pre-clinical stage and were conducted on animals or in isolated cells rather than in humans. While the results are nevertheless encouraging, it should be noted that it is far from certain that the results would be translatable to humans (Cahill et al., 2011) if and when clinical trials are conducted in humans. However, there is one other limitation, in that all the studies in this review used various extracts of OS rather than pure compounds. This could be problematic when comparing the various studies as the levels of certain secondary metabolites can vary depending on the place in which the plant is grown, even within the same country, and hence their properties can differ as well (Akowuah et al., 2004). If OS is to be developed into drugs for treating CNS disorders, either the extract properties need to be standardized or the interactions between the constituents need to be established. While there have been limited studies conducted on pure compounds of OS constituents (Erkan et al., 2008; Laavola et al., 2012; Shin et al., 2012), this however creates another issue as there could be synergistic effects between the constituents.

Besides secondary metabolites, OS is also naturally bestowed with rich primary metabolites encompassing proteins, lipids, and carbohydrates. Yet, they are not as popularly sought after, especially the OS proteins. However, the pharmaceutical demand for plant-derived proteins is tremendous, with one third of all approved pharmaceuticals being glycoproteins (Solá and Griebenow, 2010). Plant-derived proteins have emerged as a favourable therapeutic alternative mainly owing to their product safety. For instance, topical applications of plant-derived glycoproteins in humans was reported to have no significant side effects (Yao et al., 2015). Additionally, plant-based proteins eliminates potential contamination of the therapeutic drug with animal pathogens (i.e. prions, viruses, mycoplasmas) (Yao et al., 2015). Perhaps, the primary metabolites of OS such as proteins, lipids, and carbohydrates, may also hold valuable therapeutic rationale. These primary metabolites are therefore worthy of future exploration particularly in the search for a more promising treatment to a wide array of CNS disorders.

## Conclusion

The studies elaborated in current systematic review have collectively proposed that OS plays a protective role in the CNS. OS has demonstrated its ability, notably *via* its secondary metabolites (small molecules), to intercept the cross-talk between neurodegeneration, neuroinflammation, and oxidative stress, and hence contributing to neuroprotection. However, all these studies were at the pre-clinical phase and it remains to be seen if OS will be as effective in humans.

## Author Contributions

Y-SC designed, performed literature research, screened articles, and wrote the final manuscript with the help of BC. MS conceptualized, edited, and revised the final manuscript. MS, PA, and IO supervised all aspects of the research and edited the final manuscript. All authors read and approved the final manuscript.

## Funding

This work is supported by the NKEA Research Grant Scheme (NRGS), Ministry of Agriculture and Agro-Based Industry Malaysia (Grant No. NH1014D066).

## Conflict of Interest

The authors declare that the research was conducted in the absence of any commercial or financial relationships that could be construed as a potential conflict of interest.
